# Camouflage and masking behavior in adult autism

**DOI:** 10.3389/fpsyt.2023.1108110

**Published:** 2023-03-16

**Authors:** Javad Alaghband-rad, Arman Hajikarim-Hamedani, Mahtab Motamed

**Affiliations:** ^1^Department of Psychiatry, Tehran University of Medical Sciences, Tehran, Iran; ^2^Faculty of Medicine, Tehran Medical Sciences, Islamic Azad University, Tehran, Iran

**Keywords:** adult autism, camouflage, compensation, autism spectrum disorder, masking behavior

## Abstract

**Introduction:**

Autism spectrum disorder (ASD) is characterized by persistent deficits in social communication and social interaction across multiple contexts. Social camouflaging was first shown to be a characteristic of autistic persons, who actively try to disguise and compensate for their autism features in social contexts in an effort to socially blend in better. Recently, an increasing, though still insufficient, number of studies has been conducted on the concept of camouflage; however, different aspect of it, from psychopathology and etiology to its complications and consequences, are not clearly defined. We aimed to systematically review the existing literature on camouflage in autistic adults to describe the correlates of camouflage, motivations for exhibiting camouflage behavior, and the potential impacts of camouflage on the mental health of autistic individuals.

**Methods:**

We followed the Preferred Reporting Items for Systematic Reviews and Meta-Analyses (PRISMA) checklist guidelines to conduct a systematic review. The databases of PubMed and Scopus, and PsycInfo were searched for eligible studies. Studies were published between January 1st, 1980, to April 1st, 2022.

**Results:**

We included 16 articles, of which four studies were qualitative and 11 were quantitative. One study used a mix method. The assessment tools used for camouflage, the correlates of camouflage including autism severity, gender, age, cognitive profile and neuroanatomical correlates, reasons for camouflage and the impacts of camouflaging behavior on mental health are discussed in this review.

**Discussion:**

In synthesizing the literature, we conclude that camouflage seems to be more common among females who report more autistic symptoms themselves. There may also be some differences between men and women in reasons of exhibiting it and its neuroanatomical correlates. Further research is needed as to why this phenomenon is more prevalent in females with implications for gender related cognitive and neuroanatomical differences. Effects of camouflage on mental health and daily life measures of individuals such as employment, university graduation, relationship, financial status, and quality of life should be studied with more details in future studies.

## 1. Introduction

*Autism spectrum disorder* (ASD) is characterized by persistent deficits in social communication and social interaction across multiple contexts, including deficits in social reciprocity, non-verbal communicative behaviors used for social interaction, and skills in developing, maintaining, and comprehending relationships (1). This disorder is highly hereditary, with an estimated 80 to 90% heritability (2). ASD has a frequency of 16.8 per 1,000 individuals (3). Most research on ASD has focused on children; however, studies on different aspects of autism in adulthood have increased in the past two decades (4).

Besides all the commonalities, autistic individuals are a group of heterogenous individuals presenting with a variety of symptoms. This variation in symptoms might be due to differences in gender, intellectual abilities, or adaptive abilities gained throughout developmental and social life.

In addition to variability in terms of clinical presentations, many autistic individuals show a range of behaviors and strategies that help them mask some of their symptoms and mimic behaviors of neurotypical individuals in order to fit in the community. Social camouflaging was first shown to be a characteristic of autistic persons, who actively try to disguise and compensate for their autism features in social contexts in an effort to blend in socially better (5–7). Camouflaging consists of complicated copying behaviors and/or masking certain personality features with an adaptive role that aids changes to different situational demands (5, 8, 9). Camouflage is more prevalent on social occasions, although it is not limited to them (6).

Recently, an increasing, though still insufficient, number of studies have been conducted on the concept of camouflage; however, different aspects of it, from psychopathology and etiology to its complications and consequences, are not clearly defined. A growing body of evidence shows that it is a reasonably prevalent characteristic among high-functioning females with ASD. Females with ASD are more competent than males to mask their symptoms from adult observers. Being in close proximity to peer groups helped females access chances for social contact, but a deeper study indicates that girls with ASD were less likely to have the skills required to utilize those opportunities to connect with peers effectively (10).

There have been observed gender differences in the prevalence of ASD diagnosis in females (11–13). Males are diagnosed with ASD three to four times more often than females (3). Females are diagnosed with autism at a later age than boys, reducing their possibilities of obtaining care (14). In comparison to males, females with an ASD diagnosis often have more severe symptoms and comorbidities (such as intellectual disability [ID] or epilepsy) (13, 15).

Multiple biological and cognitive factors seem to explain the disparities between men and girls in terms of ASD diagnosis (5), such as: (a) cognitive development disparities exist since females seem to have superior visual abilities and higher IQ scores (8); (b) the fact that females are preserved by intrinsic systems, such as sex-steroid hormones (16); (c) variations in empathizing and systemizing, whereby females seem to be more sympathetic due to social compensating abilities, and/or (d) the camouflage of ASD core symptoms (6). In accordance, the female phenotype of ASD is characterized by the capacity to conceal autistic symptoms. Females are likely more socially adept due to their ability to camouflage autistic symptoms, which may lead to a delayed or missing diagnosis (8). Though the underlying etiology is not attributed to a specific factor but differences in social norms and expectations from males and females, the difference in the autism severity in men and women, and neuropsychological differences have all been suggested.

Camouflage has been demonstrated and evaluated using various measures in the literature. Early evaluations have emerged from qualitative descriptions of autistic individuals, their families, and mental health providers working with them. Later works focused on quantifying camouflage regarding the discrepancy between individuals’ autistic traits and their observed behaviors. The most recent quantifying measures are self-report questionnaires which give a score for the extent of camouflage behavior one employs. Hull, Laura, et al. created the Camouflaging Autistic Traits Questionnaire (CAT-Q) to measure the extent to which autistic and non-autistic adults engage in three aspects of social camouflaging: (1) “compensation” for autism-related difficulties in social situations, such as using scripts and copying others from carefully observing other people; (2) “masking” one’s autistic characteristics, by constantly monitoring one’s own behaviors (e.g., eye contact, facial expression, gesture) to show a non-autistic persona to others; and (3) “assimilation” which describes behavioral techniques used to fit in better with others (e.g., forcing oneself to interact by performing and pretending) (17).

Although the effect of camouflage is understudied, this concept has been referred to in the literature as imitation (18), copying (8, 19, 20), masking (19–21), and compensation (21). Camouflage may impose many difficulties on autistic individuals, including depression, anxiety, and burnout. Additionally, it may also lead to delayed diagnosis, which prevents them from getting appropriate care.

Not a large number of systematic reviews have been conducted on camouflage. Three of the previous reviews studied camouflage in females only (7, 22). The most recent systematic review by Cook et al. included studies done on both males and females. However, the review is not specified to the adult population (23).

We aimed to systematically review the existing literature on camouflage in autistic adults to describe the correlates of camouflage, motivations for exhibiting camouflaging behavior, and the potential impacts of camouflage on the mental health of autistic individuals. We evaluated the strengths and weaknesses of the research base and highlighted the areas where the evidence is consistent and reliable while identifying wherever the evidence is inconsistent.

## 2. Materials and methods

### 2.1. Instrument

**Figure 1:**
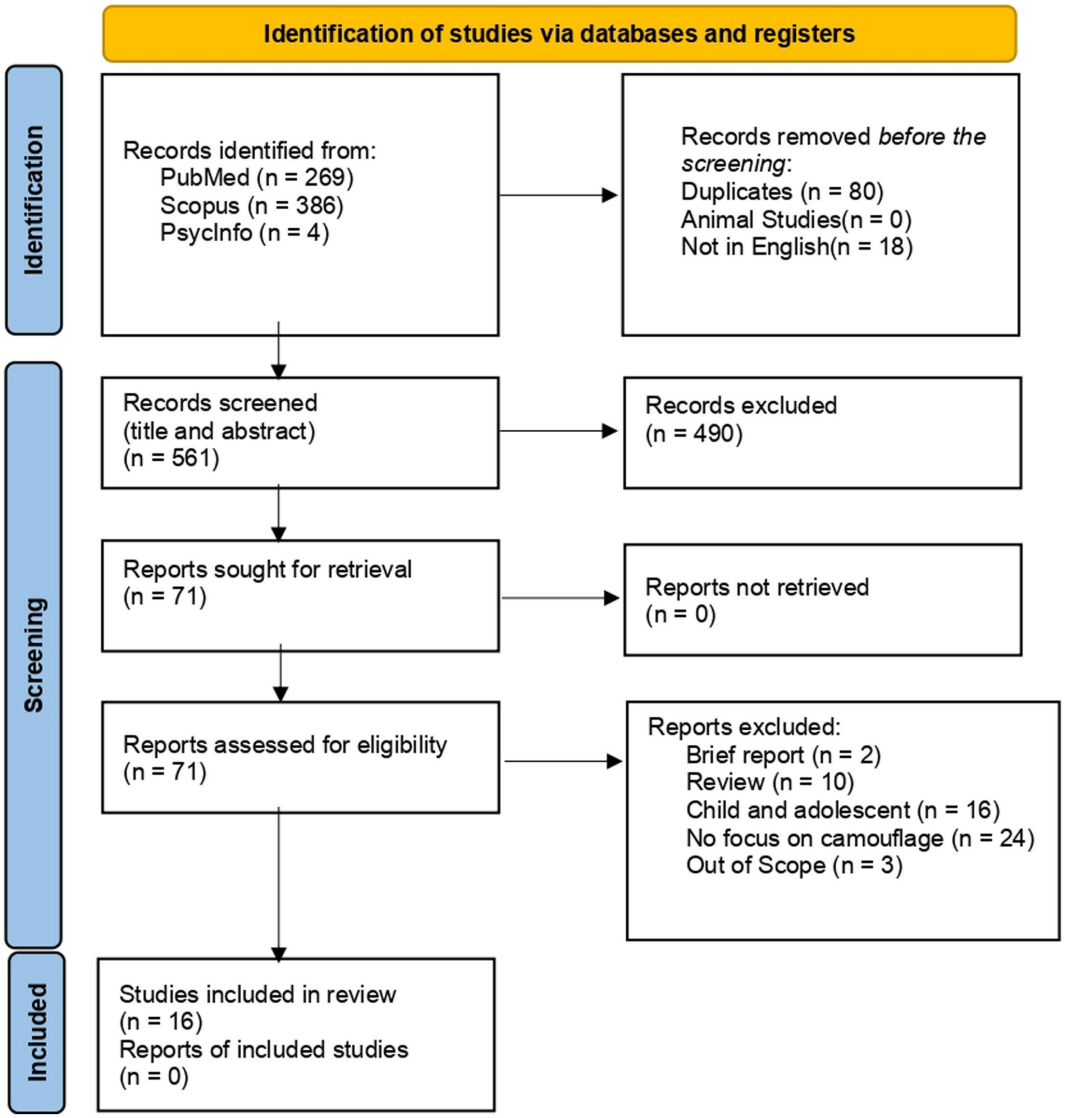
Study selection. Preferred items for Systematic Reviews and Meta-Analyses (PRISMA) flow diagram. Out of 659 identified studies and after the application of the inclusion and exclusion criteria, 16 studies were included in the synthesis (25).

We followed the Preferred Reporting Items for Systematic Reviews and Meta-Analyses (PRISMA) (24, 25) checklist guidelines to conduct a systematic review. The following databases (PubMed, Scopus, and PsycInfo) were chosen for research (Figure 1).

### 2.2. Inclusion and exclusion criteria

Before the screening, the following inclusion and exclusion criteria were established. Inclusion criteria were as follows: Human studies only, measurements from a minimum of one established cognitive test and English as the language of publication. Exclusion criteria were as follows: studies with participants under the age of 18, letters, editorials, reviewers, and commentaries.

### 2.3. Search strategy

The databases of PubMed and Scopus, and PsycInfo were searched for eligible studies. Studies were published between January 1st, 1980 to April 1st, 2022.

Separate search strategies were created for each database. The search terms we used to mine each thesaurus began with a mix of generic terms drawn from cognitive domains and terms drawn from our own prior knowledge. Several preliminary scoping searches were undertaken, from which a handful of essential search phrases were derived. Listed below is an example of a conclusive search method for the PubMed database.

(“adult*”[All Fields] OR (“grown”[All Fields] AND “up”[All Fields]) OR (“men”[MeSH Terms] OR “men”[All Fields] OR “man”[All Fields])) AND (“autism s”[All Fields] OR “autisms”[All Fields] OR “autistic disorder”[MeSH Terms] OR (“autistic”[All Fields] AND “disorder”[All Fields]) OR “autistic disorder”[All Fields] OR “autism”[All Fields] OR (“autism spectrum disorder”[MeSH Terms] OR (“autism”[All Fields] AND “spectrum”[All Fields] AND “disorder”[All Fields]) OR “autism spectrum disorder”[All Fields])) AND (“camouflag*”[All Fields] OR “compensat*”[All Fields] OR (“mask s”[All Fields] OR “masked”[All Fields] OR “masking”[All Fields] OR “masks”[MeSH Terms] OR “masks”[All Fields]))

The words enclosed by brackets indicate whether the term is a search term or a Medical Subject Heading (MeSH). All search queries were examined for registration as MeSH terms.

Whether this was the case, a search was run to see if it was essential, as indicated, to add the search phrase booth as a mesh term with explosion and a search term.

This was done to reduce the complexity of the search phrase while maintaining the same search results since certain databases struggled to process lengthy search strings. Similar experiments were done in all datasets.

### 2.4. Data screening

All references were imported into EndNote 20, where duplicates were subsequently discovered and eliminated.

### 2.5. Data charting process

One reviewer collaboratively devised a data-charting form to select which variables to extract. The reviewer extracted data separately and discussed the findings in the event of a dispute. Article characteristics (author, year, country, journal, and citations) and population characteristics (autism severity, autism diagnostic tools) were extracted, along with sample size (n) and mean age and sex. Research parameters (intervention, outcome, and impact) were used for the conclusion of data extraction.

### 2.6. Quality assessment

The risk of bias was evaluated using a technique developed by The Effective Public Health Practice Project (EPHPP) [21] to assess the methodological quality of primary studies using a range of research designs. There are six component ratings: (1) selection bias, (2) study design, (3) confounders, (4) blinding, (5) data collection method, and (6) withdrawals and dropouts. Each element is ranked as weak, moderate, or strong. If there are no weak ratings, the international rating is strong; if there is one poor rating, it is moderate; and if there are two or more weak ratings, it is weak. The tool was adjusted somewhat since the component domain (c) “confounders” was particularly applicable to randomized controlled research, where control for group differences is crucial. In the current research, the populations serve as their own controls, making this factor less significant. Again, the two reviewers evaluated the research separately and discussed the findings in the case of disagreement.

## 3. Results

### 3.1. Type of studies

We included 16 articles, of which four studies were qualitative, and 11 were quantitative were included in this review. One study used a mixed method. Detailed characteristics and main findings of the studies are demonstrated in Table 1.

**Table 1:** Characteristics and main findings of studies included in the systematic review.

Author^ref^	Year	Country	Sample size (*n*)		Age range	Sex (%male)	Camouflage measure	Gender difference	Main findings
			Autistic (%male)	Non-autistic (%male)					
Dell’Osso et al. (26)	2021	Italy	2,141		University students	33.90	CAT-Q	No	Higher camouflage in higher autistic traits
			–	–					
									Higher camouflage in “pure science” students
Hull et al.(27)	2021	United Kingdom and United States	305		18–75	36.49	CAT-Q	No	Camouflage was associated with more generalized anxiety, depression, and social anxiety
			305(36.5)	0					
Belcher et al. (28)	2021	United Kingdom	80		18–40	50	CAT-Q	No	Autistic people scored higher in CAT-Q, but camouflage did not predict first impressions or Age at diagnosis
			40(50)	40(50)					
Beck et al. (29)	2020	United States	156		18–42	Only females	CAT-Q	–	High CAT-Q camouflaging scores were related to suicidality, psychological distress, and impaired functioning
			18(0)	40(0)					
Hull et al.(30)	2020	United Kingdom	778		Average = 34.56	40.95	CAT-Q	Higher camouflage in autistic females	Autistic females consistently achieved greater camouflage ratings than autistic males. No difference between non-autistic males and females
			306(35.3)	472(40.9)					
Cassidy et al.(31)	2020	United Kingdom	160		18–23	13.12	CAT-Q	–	Camouflage may increase the sense of rejection and the risk of suicidal actions and thoughts
			–	–					
Livingston et al. (32)	2020	United Kingdom	117		18–77	18.80	Compensation checklist	No	Higher compensation scores in autistic individuals and individuals with higher education
			58(24.1)	59(13.5)					
Schuck (33)	2019	United States	62		18–55	58.06	Subtracting ADOS from AQ score	Higher camouflage in females	Negative correlation between camouflage and emotional expressivity in females
			28(60.7)	34(55.9)					
Lai et al. (34)	2019	Cyprus, Taiwan, United Kingdom, Canada	119		18–42	52.10	Subtracting ADOS from AQ score	Higher camouflage in females	Increasing camouflaging was associated with heightened ventromedial prefrontal cortex self-representation response in females.
			57(50.8)	62(53.2)					
Hull et al.(17)	2018	United Kingdom and Canada	832		16–82	40.87	CAT-Q	–	The CAT-Q is a reliable and valid self-report measure of social camouflaging in autistic and non-autistic male and female populations
			354(30.5)	478(40.2)					
Lai et al. (35)	2017	Taiwan, Canada, Cyprus, and United Kingdom	60		18–49	50	Subtracting ADOS from AQ score subtracting ADOS from RMET score	Higher camouflage in autistic females	Higher camouflage score in women than men
			30(50)	0					
									Greater camouflaging was associated with more depressive symptoms in men and better signal-detection sensitivity in women with autism
Cook et al. (36) Qualitative study	2022	United Kingdom	17		24–63	75	Self -reflection on recorded videos	–	Camouflage behaviors included masking, innocuous behavior, modeling neurotypical communication, and active self-presentation
			17(35.3)	0					
Bradley et al.(37) Qualitative study	2021	United Kingdom	277		Average = 38.25	33.57	–	–	Reported dangers of camouflage were exhaustion, the creation of unreal perceptions, and mental problems. Positive aspects are protection and resiliency.
			277(33.6)	0					
Schneid and Raz(38) Qualitative study	2020	Israel	24		16–55	76.92	–	–	Camouflage is used to manage social impressions; however, it feels artificial.
			24(76.9)	0					
Cage and Troxell-Whitman(39) Mix method study	2019	United Kingdom	262		18–66	42.36	CAT-Q	Females exhibit camouflage for more conventional reasons compared to men	Camouflage was correlated with anxiety and stress but not depression. Females exhibit camouflage for more conventional reasons compared to men. Causes for camouflage were fitting to the neurotypical community, avoiding bullying, and internalized stigma.
			262(42.4)	0					
Hull et al. (40) Qualitative study	2017	United Kingdom	92		18–79	32.60	–	–	Camouflage is motivated by fitting in and making connections. Short-term camouflaging causes significant exhaustion and anxiety; In the long term, it affects mental health, self-perception, and access to care.
			92(32.6)	0					

CAT-Q, Camouflaging Autistic Traits Questionnaire; AQ, Autism Quotient; ADOS, Autism Diagnostic Observation Schedule; RMET, Reading the Mind in the Eyes Test.

### 3.2. Camouflage assessment tools

Camouflage was assessed using different measures. The most common tool (eight studies) was the Camouflaging Autistic Traits Questionnaire (CAT-Q) (26–31, 36, 39) which is a 25-item self-report questionnaire developed in 2018 by Hull et al. (17). One study utilized a 31-item Compensation Checklist to evaluate strategies including masking, shallow compensation, deep compensation, and accommodation in participants (32).

In some studies, camouflage was scored by subtracting Autism Diagnostic Observation Schedule (ADOS) score from Autism Quotient (AQ) score (33–35). In a number of studies screening and open questions were used to assess the extent of camouflaging (37–40). In a study by Cook et al. authors asked the participants to watch their own interactions and self-reflect on camouflaging (36).

### 3.3. Correlates of camouflage

Some correlates have been suggested for camouflage in a number of studies (26, 33).

#### 3.3.1. Autism severity

Camouflaging was reported to be higher in autistic individuals compared to non-autistic individuals (28, 30). A significant positive relationship between camouflaging behavior and autism severity (higher scores in autism questionnaires) was observed in some studies (26, 28, 31, 32). Dell’Osso et al. reported a strong association between autistic traits and camouflaging in university students (26). Higher AQ10 scores and having an autism diagnosis were positively associated with total compensation and shallow compensation (32). Similarly, a significant positive relationship was found between AQ28 and CAT-Q total and subscales scores in 160 undergraduate students in the United Kingdom (31).

#### 3.3.2. Gender

It has been revealed by three studies that camouflage behavior is more common in autistic females rather than males (30, 33, 35). However, some studies could not find the gender difference in the non-autistic population (26, 28, 30) or in samples of autistic adults (27, 28). Using a Compensation Checklist, Livingston et al. did not find a relationship between compensation score and sex (32).

In addition to the prevalence of camouflage behavior, a gender difference was found in the reasons for camouflaging in autistic women and men (39).

#### 3.3.3. Education

Students of pure sciences reported higher camouflaging behavior compared to applied sciences (26). This group scored higher in The Adult Autism Subthreshold Spectrum (AdAS). Livingston et al. reported that a higher educational level was associated with higher compensation in autistic and non-autistic adults (32).

#### 3.3.4. Cognitive profile

One study showed a negative correlation between emotional expressivity and positive expressivity in autistic females; however, this correlation was not observed in autistic males (33). In this study, no correlation between working memory and camouflage was found. No significant correlation between IQ and camouflage was found by Lai et al. (35); However, the authors found a positive correlation between camouflage and signal detection sensitivity. Similarly, Belcher et al. reported no correlation between camouflage and executive function or theory of mind in 80 autistic participants (28).

#### 3.3.5. Age and age at diagnosis

Only one study explored the effect of camouflage on the Age of receiving ASD diagnosis and found no relationship between them (28). No correlation was found between Age and camouflage in two other studies (32, 35). However, a negative correlation between the compensation subscale of CAT-Q and Age was reported in undergraduate students in one study (31).

#### 3.3.6. Neuroanatomical correlates of camouflage

Only two studies explored camouflaging in relation to neuroanatomical regions or activity. One study examined the neural responses during mentalization and self-representation and found a positive correlation between camouflage and activation of the ventromedial prefrontal region during self-representation (34). Lai et al. revealed a gender difference in neuroanatomical correlates of camouflage. While no neuroanatomical association with camouflage was found in men, smaller volumes in the medial temporal and cerebellum were associated with higher camouflaging in autistic women (35).

### 3.4. Reasons and motivations for camouflage

Some reasons mentioned for camouflaging by autistic individuals included helping them to access the social world and social opportunities and making friends, being accepted by others, maintaining safety, and building resiliency (37, 40). Different reasons for camouflage were described in one study; while women engage in camouflage to serve a functional purpose in the workplace or education, men camouflage to be more comfortable in social interactions (39).

### 3.5. Impacts of camouflage behavior on mental health

Some studies reported the consequences of camouflage in autistic and neurotypical individuals (27, 29, 35). According to a study in 2020, camouflaging was a predictor of psychological distress (measured by The Depression Anxiety Stress Scales 21) and functional challenges (measured by The World Health Organization Disability Assessment Schedule, Second Edition) but not suicidality in women. Though in the high camouflage group, a significant relationship between CAT-Q score and suicidality was found (29). Cassidy et al. found an increased risk of experiencing thwarted belongingness and lifetime suicidality in undergraduate students with higher camouflaging autistic traits (31).

In a similar vein, Hull et al. demonstrated an association between camouflage and generalized anxiety, social anxiety, and to a smaller extent between camouflage and depression in a sample of 305 autistic adults (27). The effect of camouflage on anxiety and stress was replicated in the study of Cage and Troxell-Whitman (39). Lai et al. reported a correlation between depression and camouflage in autistic men but not women. Such correlation was not observed for anxiety (35).

Moreover, the impacts of camouflage have been investigated through open questions about the experience of autistic individuals and further thematic analyses (37, 40). Exhaustion, depression, and anxiety, making unreal perceptions of others were the consequences stated by autistic adults. For some individuals, camouflaging affected their self-perception and gave them a feeling of deception, which led to anxiety, isolation, and a sense of alienation (38, 40).

## 4. Discussion

We did a systematic review of empirical studies of camouflage phenomena in autistic adults. Social camouflage is an emerging topic of research with an increasing number of studies over the last decade. Camouflage research has also included various concepts of masking, compensation, copying, or imitation in different studies (23, 26).

Recent attempts to develop an index (33, 34) and questionnaires (17, 32) with gender-sensitive criteria for camouflage have made this field of research more interesting. Almost all the measures are self-reports through questionnaires. In one study, autistic individuals were asked to report camouflage behaviors when watching their own video recordings (36). Assessing camouflage with objective measures is not studied and needs further attention. In one study, autistic individuals were asked to report camouflage behaviors when watching their own video recordings. It should be noted that some autistic individuals might engage in camouflaging while they are not aware of it and do not report it, respectively.

In synthesizing the literature, we conclude that camouflage seems to be more common among females who report more autistic symptoms themselves (26, 28, 32) and are perhaps more aware of their challenges in social situations. This self-awareness and continuous effort to mask their symptoms would naturally lead to more negative emotional outcomes (27, 29).

There are various correlates of camouflage that makes it more interesting, as follows:

### 4.1. Gender differences

There are a number of studies suggesting camouflage to be a characteristic of females with ASD or reporting it to be more common among this group of female patients (30, 33, 35). It is noteworthy to mention that not all the studies found such a difference.

### 4.2. Cognitive profiles

A number of studies evaluated cognitive profiles of adult ASD, and a subset of these studies looked into those patients who present with camouflaging (28, 33, 35). Higher vocal expression, behavior involvement in social behavior (41), and higher education (32) have been reported in these studies. There are also studies that found those with camouflaging reporting more autistic symptoms (26).

### 4.3. Neuroanatomical findings

There are very few studies reporting on neuroanatomical correlates of camouflaging. In those reports, activation of the ventromedial prefrontal region and smaller volumes of medial temporal and cerebellum were associated with higher camouflaging.

As it is described, the correlates of camouflage are not widely studied, and there exist many inconsistencies, and many questions have remained unanswered.

Not many studies explored the reasons and motivations for camouflage. In general, autistic individuals use camouflage as a tool to help them to fit better in neurotypical communities (37, 40). Further qualitative studies about the lived experiences of autistic individuals about different types of camouflaging, the situations which provoke camouflaging, and the objectives that they seek when they camouflage would be warranted to make the reasons behind camouflaging more clear.

The impacts of camouflaging on the mental health of autistic individuals have been described in some studies. It can be summarized that although camouflage could provide some advantages to better fit in society can lead to some disadvantages like anxiety, depression, and exhaustion. Kim et al. conducted a meta-regression analysis of correlates of quality of life in autistic individuals (42). They did not find a significant effect size for IQ or autism severity. Nevertheless, they found a significant effect size for social functioning and quality of life. They suggested that camouflaging can have positive impacts on social functioning as individuals can reach their short-term goals by using camouflage. However, the short-term benefits of camouflage should be weighed against the long-term costs of camouflage, such as depression and exhaustion. Accordingly, the impacts of camouflage on the quality of life of autistic individuals should be considered in a multilayered and deeper view. Effects of camouflage on daily life measures of individuals such as employment, university graduation, relationship, financial status, and quality of life should be studied with more detail in future studies. The impact of camouflage on service delivery, misdiagnosis, and delayed diagnosis are other understudied areas that should be addressed when exploring camouflage in autism (43).

In conclusion, camouflage is apparently a set of strategies and self-control skills (44) used by autistic females who are more self-aware of their autistic symptoms (28, 32). This also explains why camouflage correlates positively with university education. These continuous efforts to mask symptoms are mentally costly for them and lead to mental health challenges (27, 37, 40). Further research is needed as to why this phenomenon is more prevalent in females, with implications for gender-related cognitive and neuroanatomical differences. Camouflaging is also important clinically, as health professionals are not familiar enough to diagnose adult ASD, particularly those who camouflage. Better understanding of the phenomenon would decrease the long-term consequences of non-diagnosis or misdiagnosis in this population.

## Data availability statement

The raw data supporting the conclusions of this article will be made available by the authors, without undue reservation.

## Author contributions

JA-r contributed in idea creation, design of the study, categorizing and synthesizing the data, and drafting. AH-H contributed in idea creation, databases search, categorizing the data, and drafting. MM contributed in idea creation, design of the study, databases search, categorizing and synthesizing the data, and drafting. All authors contributed to the article and approved the submitted version.

## Conflict of interest

The authors declare that the research was conducted in the absence of any commercial or financial relationships that could be construed as a potential conflict of interest.

The reviewer EC declared a past collaboration with author JA-r to the handling editor.

## Publisher’s note

All claims expressed in this article are solely those of the authors and do not necessarily represent those of their affiliated organizations, or those of the publisher, the editors and the reviewers. Any product that may be evaluated in this article, or claim that may be made by its manufacturer, is not guaranteed or endorsed by the publisher.
